# Identification novel prognostic signatures for Head and Neck Squamous Cell Carcinoma based on ceRNA network construction and immune infiltration analysis

**DOI:** 10.7150/ijms.53531

**Published:** 2021-01-19

**Authors:** Haiting Zhou, Yi He, Lingling Li, Cheng Wu, Guoqing Hu

**Affiliations:** 1 Department of Oncology, Tongji Hospital, Tongji Medical College, Huazhong University of Science and Technology, Wuhan, Hubei, 430030, P.R. China.; 2 Department of Orthopedics, Tongji Hospital, Tongji Medical College, Huazhong University of Science and Technology, Wuhan, Hubei, 430030, P.R. China.

**Keywords:** head and neck squamous carcinoma, ceRNA network, immune infiltration, prognostic signature, nomogram

## Abstract

**Background:** Head and neck squamous cell carcinoma (HNSCC) is a common malignancy with high mortality and morbidity worldwide, but the underlying biological mechanisms of molecules and tumor infiltrating-immune cells (TIICs) are still unknown.

**Methods and Results:** We obtained mRNAs, lncRNAs, and miRNAs expression profiles of 546 HNSCC from The Cancer Genome Atlas (TCGA) database to develop a ceRNA network. CIBERSORT was employed to estimate the fraction of 22 types of TIICs in HNSCC. Univariate and multivariate Cox regression and lasso regression analyses were used to develop prognostic signatures. Then, two novel risk signatures were constructed respectively based on six ceRNAs (ANLN, KIT, PRKAA2, NFIA, PTX3 and has-miR-148a-3p) and three immune cells (naïve B cells, regulatory T cells and Neutrophils). Kaplan-Meier (K-M) analysis and Cox regression analysis further proved that these two signatures were significant prognostic factors independent of multiple clinicopathological characteristics. Two nomograms were built based on ceRNAs-riskScore and TIICs-riskScore that could be used to predict the prognosis of HNSCC. Co-expression analysis showed significant correlations between miR-148a-3p and naive B cells, naive B cells and plasmas cells.

**Conclusion:** Through construction of the ceRNA network and estimation of TIICs, we established two risk signatures and their nomograms with excellent utility, which indicated the potential molecular and cellular mechanisms, and predicted the prognosis of HNSCC.

## Introduction

Head and neck squamous cell carcinoma (HNSCC) ranks the sixth common malignancy, with more than 500 000 new cases diagnosed worldwide each year 1.

HNSCC mainly arises from larynx, oropharynx, oral cavity and hypopharynx. Smoking, drinking alcohol and infection with human papillomavirus (HPV) are the main identified risk factors for HNSCC patients 2-5. Most patients of HNSCC are in the mid-late stage due to the anatomic factors and weak consciousness of self-care. Although the development of treatment strategies including surgical operation, radiotherapy, chemotherapy and targeted therapy, the 5-year overall survival rate of locally advanced HNSCC has no significant improvement 6. Therefore, it is in a desperate need to look for new molecular and cellular biomarkers to better guide diagnosis and treatment of HNSCC.

In 2011, Salmena et al. presented the ceRNA hypothesis, in which microRNAs (miRNAs) act as “sponges” sharing common miRNA recognition elements (MREs) with both coding and non-coding RNAs to regulate their respective expression level 7. Long non-coding RNAs (lncRNAs) are endogenous RNA molecules greater than 200 nucleotides in length and lack protein-coding ability 8. Importantly, lncRNAs competitively bind to miRNAs, so as to regulate the expression level of mRNAs and involve in the regulation of biological behaviors of tumor cells. Many studies have demonstrated that lncRNAs play an essential part in the occurrence and progression of various malignancies 9-11. As critical negative regulators of gene expression, miRNAs are small, non-coding RNAs, with approximately 22 nucleotides 12. Numerous studies showed that miRNAs affect gene expression by guiding RNA-induced silencing complex (RISC) to target mRNA, leading the degradation or translation inhibition of RNA 13.

Recently, tumor immune-infiltrating cells (TIICs) have attracted widespread attention, especially in light of significant progress in immunotherapy. HNSCC is abundant with TIICs, and the majority of patients respond positively to immunotherapy 14. Some studies have shown that different composition and localization of TIICs were firmly related to the prognosis of HNSCC 15. However, in the past, researchers used traditional methods such as immunohistochemistry and flow cytometry to explore the composition of immune cells in tumors, which set limit on the number of cells that could be synchronously examined. CBERSORT is a new method based on machine learning, which enables estimation of 22 immune cell types' abundances from gene expression profiles of various bulk tissues 16. For the superior performance of CIBERSORT, numerous studies performed it to analyze the fraction of immune cells in multiple cancers.

The roles of ceRNA networks and TIICs in HNSCC have been studied respectively 17, 18. Up to now, no comprehensive study integrating the function of both ceRNAs and TIICs in HNSCC has been published. In the current study, we identified differentially expressed ceRNAs of HNSCC using transcriptome profiles retrieved from the TCGA database. Through CIBERSORT, we analyzed the proportion of 22 immune cells in HNSCC. Furthermore, two prognostic risk signatures based on survival-related ceRNAs and significant TIICs were established. Most strikingly, we performed co-expression analysis between ceRNAs and TIICs to identify underlying immune-related biomarkers.

## Materials and methods

### Data collection

Transcriptome profiling (level 3) of 546 HNSCC samples was collected from TCGA-GDC database (https://portal.gdc.cancer.gov/) (workflow Type: HTSeq-Counts, Project: TCGA-HNSC), including RNA-seq and miRNA-seq data. Corresponding clinical information was obtained from the database as well. Patients with follow-up time under 30 days and incomplete follow-up data were eliminated. Transcriptome profiles of 502 tumor samples and 44 normal samples were obtained. And 519 patients with follow-up data were used for further survival analysis. All data was preprocessed by Perl language and R software. All data were downloaded from the TCGA database; no additional approval by the Ethics Committee was claimed.

### Analysis of differentially expressed mRNAs (DEGs), miRNAs (DEMs) and lncRNAs (DELs)

Ensemble database (http://asia.ensembl.org/index.html) was used to annotate mRNA and lncRNA. MiRBase database (http://www.mirbase.org/) was applied to annotate miRNA^19^. All transcriptome profiling data was normalized by Voom standardized method. Differential expression analyses of mRNA, miRNA and lncRNA between HNSCC and adjacent tissues were realized by “DESeq2” package with R software. All P values were adjusted by false discovery rate (FDR). Setting adjusted P -value < 0.01 and |log (Fold change) | >1 as the filter criteria. We then drew heatmaps and volcano figures for DEGs, DEMs and DELs with the R package “ggplot2”. Gene ontology (GO) and Kyoto Encyclopedia of Genes and Genomes (KEGG) enrichment analyses of DEGs were realized via R software with clusterProfiler, enrichplot and ggplot2 packages, and both p- and q-value < 0.05 were considered significantly enriched.

### Construction of the ceRNA network

“GDCRNATools” package 20 in R software was utilized to establish a ceRNA network. We chose Starbase (http://mirtarbase.mbc.nctu.edu.tw/) to predict the interactive relationship between DEGs and DEMs or DELs and DEGs, which comprehensively identify the RNA-RNA and protein-RNA interaction networks based on 108 CLIP - Seq datasets of 37 independent studies 21. Afterwards, we chose miRNA regulated both mRNAs and lncRNAs with significant results in hypergeometric test and correlation analysis to establish a ceRNA network. The network was then visualized by Cytoscape v.3.5.1 22.

### Construction and evaluation of ceRNAs-related prognostic signature

We performed the least absolute shrinkage and selection operator (LASSO) analysis to control overfitting using “glmnet” package in R software. Multivariate Cox regression analysis was applied for selecting optimal biomarkers to construct a ceRNAs-related signature. This signature employed stepwise selection and selected an optimal model by Akaike information criteria (AIC). Mann-Whitney U test and Kruskal-Wallis test were utilized to analyze the relationship between clinicopathological characteristics and the risk score. Then we employed K-M method to explore the survival variations between high-risk group and low-risk group. Afterwards, univariate and multivariate Cox analyses were performed to further explore the prognostic value of the riskScore independent of other clinical features involving age, gender, grade and TNM stage. Next, a nomogram was established to predict the survival possibility of each patient. Through calibration, ROC (receiver operating characteristic) and K-M curves, we evaluated the accuracy and discrimination of the nomogram.

### Estimation of TIICs fractions

CIBERSORT (http://cibersort.stanford.edu/) is a deconvolution algorithm to precisely estimate proportions of multiple immune cells in gene expression profiles from bulk tumors. The continuous development of CIBERSORT raised a growing focus on the studies of cellular heterogeneity 23, 24. To explore the cellular reasons for biological mechanisms of the significant genes in the ceRNA network, our current study estimated 22 types of TIICs in HNSCC and adjacent tissues via CIBERSORT algorithm. Only when the samples with P-value of CIBERSORT less than 0.05 could be used for further survival study. The bar plot and the heatmap were used to describe the proportion of 22 TIICs in each sample. Then, Wilcoxon rank-sum test was applied to assess the difference of TIICs between tumor and normal tissues. The results were visualized via violin plot.

### Construction and evaluation of TIICs-related prognostic signature

Univariate Cox analysis was performed to find prognostic TIICs. Lasso regression was utilized to shrink TIIC candidates. Then significant TIICs were put into multivariate model to construct a TIICs-related prognostic signature. Univariate and multivariate Cox analyses were utilized to explore independent prognostic factors for HNSCC. ROC and K-M curves were used to evaluate the predictive and prognostic value of the signature. Then, we constructed a nomogram to predict the prognosis of HNSCC. And the calibration curve was utilized to access the accuracy. Pearson correlation analysis was applied for investigating the association between each type of TIICs and significant ceRNAs.

### Statistical analysis

All statistical analyses were performed with R software (v4.0.2) (package: GDCRNATools, ggplot2, DEseq2, clusterProfiler, survminer, survival, glmnet, timeROC, rms, preprocessCore, pheatmap, corrplot and vioplot).

## Results

### Identification of DEGs, DEMs and DELs

**Figure 1:**
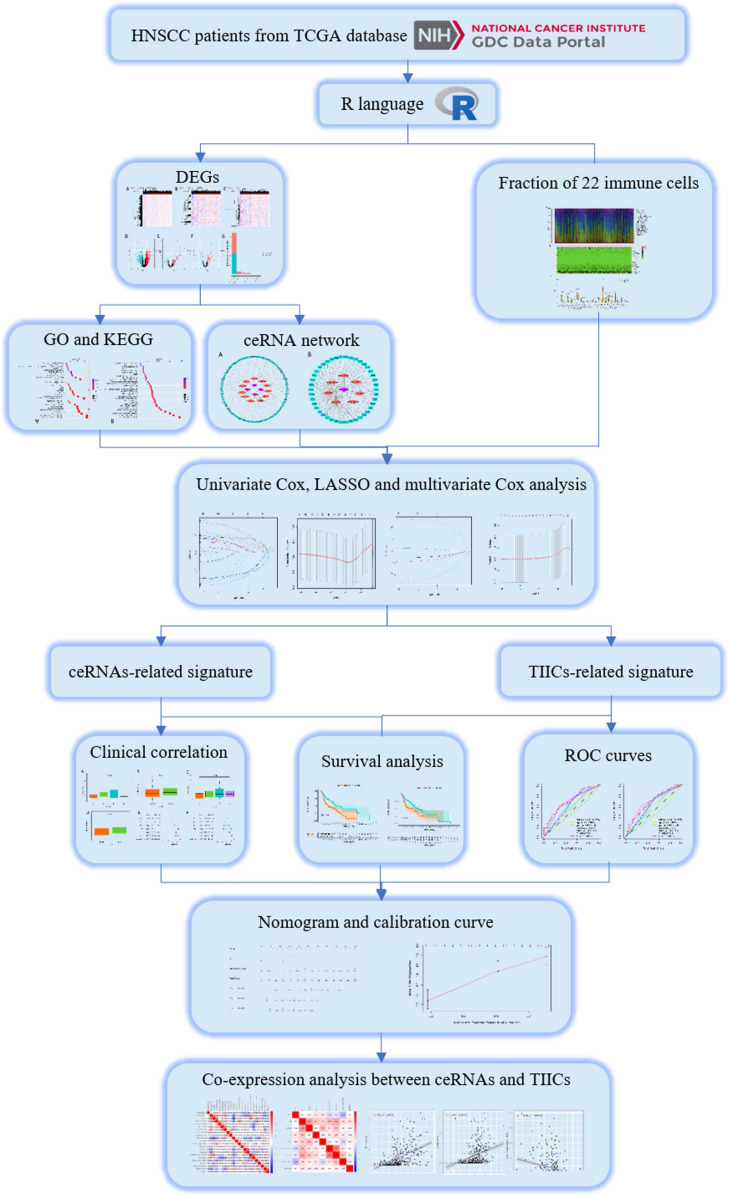
A flow chart of the analytical process.

**Figure 2:**
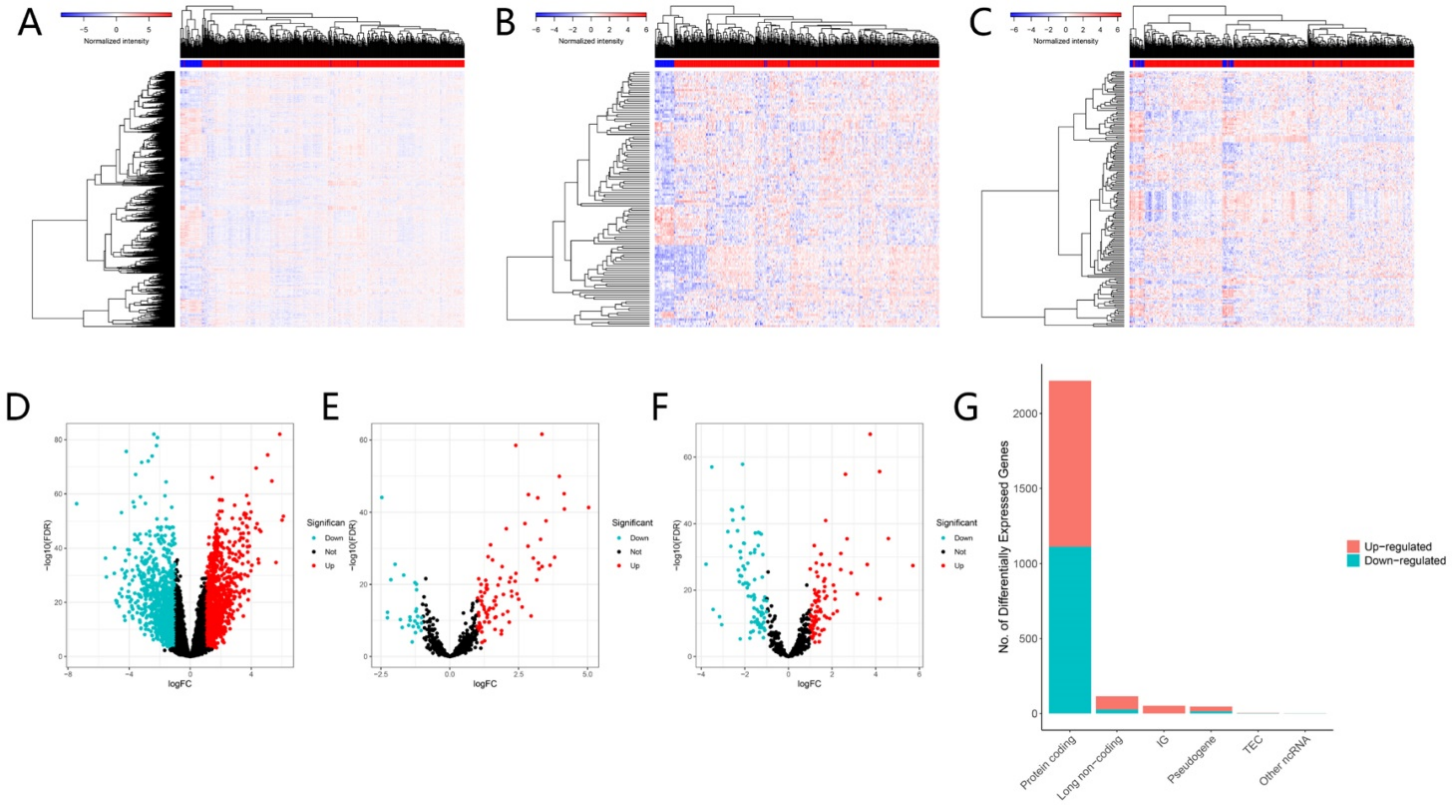
Differentially expressed genes between normal and tumor tissues. The heatmap (A) and the volcano plot (D) of 2219 DEGs; The heatmap (B) and the volcano plot (E) of 115 DELs. The heatmap (C) and the volcano plot (F) of 166 DEMs; The composition of differentially expressed genes (G). LogFC > 1.0 or < -1.0 and FDR < 0.01.

The flow chart of our work is shown in** Figure 1**. We integrated gene expression profiles of 502 tumor samples and 44 normal samples from TCGA into this study. And 2219 DEGs (1106 up-regulated, 1113 down-regulated) and 115 DELs (89 up-regulated, 26 down-regulated) were acquired between normal samples and tumor samples. In order to construct a mRNA-miRNA-lncRNA ceRNA network, we also integrated miRNA expression profiles of 569 samples of HNSCC patients. As a consequence, 166 DEMs (83 up-regulated, 83 down-regulated) were retrieved with the same cut-off value (**Fig. 2A-G**).

### GO and KEGG Enrichment Analysis

**Figure 3:**
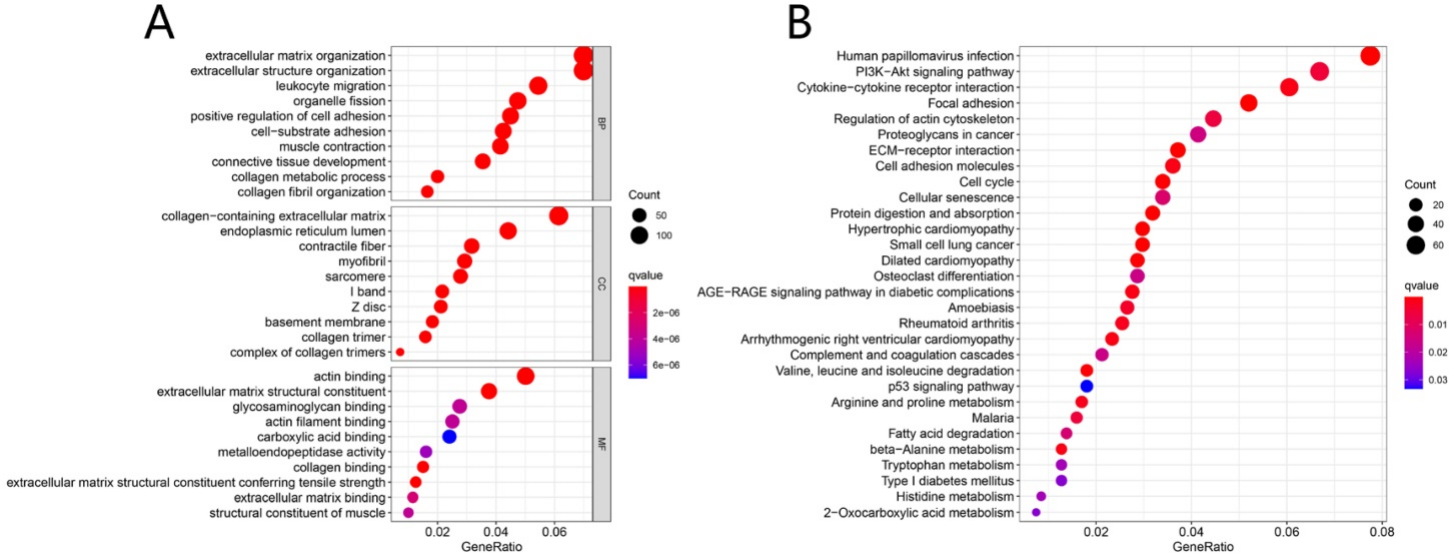
Functional enrichment analysis. GO analysis of DEGs in HNSCC (A); KEGG pathways of DEGs in HNSCC (B).

Results from GO enrichment analysis manifested that the DEGs almost mapped to extracellular matrix organization, extracellular structure organization, leukocyte migration, organelle fission and positive regulation of cell adhesion (**Fig. 3A**). The KEGG enrichment analysis displayed the enrichment of Human papillomavirus infection, PI3K-Akt signaling pathway, Cytokine-cytokine receptor interaction, Focal adhesion, Regulation of actin cytoskeleton and other tumor-related signaling pathways (**Fig. 3B**).

### The ceRNA network construction and survival analysis

**Figure 4:**
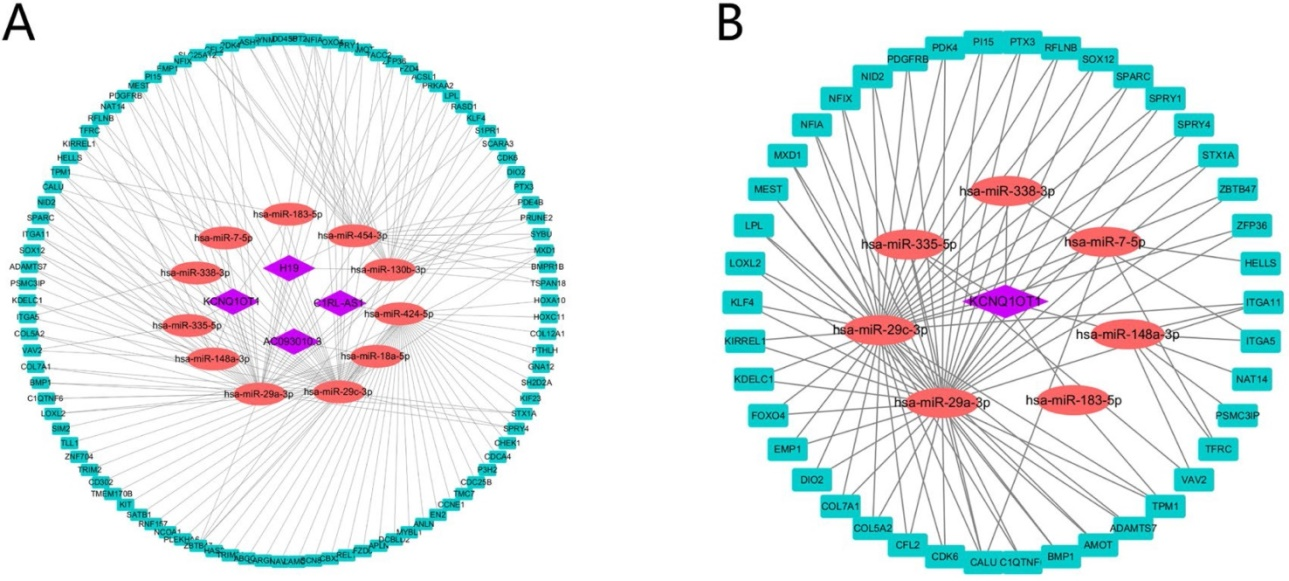
Construction of the ceRNA network. The ceRNA network of DEGs, DEMs and DELs (A). The lncRNA KCNQ1OT1 sub-network (B). The rectangles indicate mRNAs in light blue, ellipses represent miRNAs in light red and diamonds represent lncRNAs in light purple.

**Figure 5:**
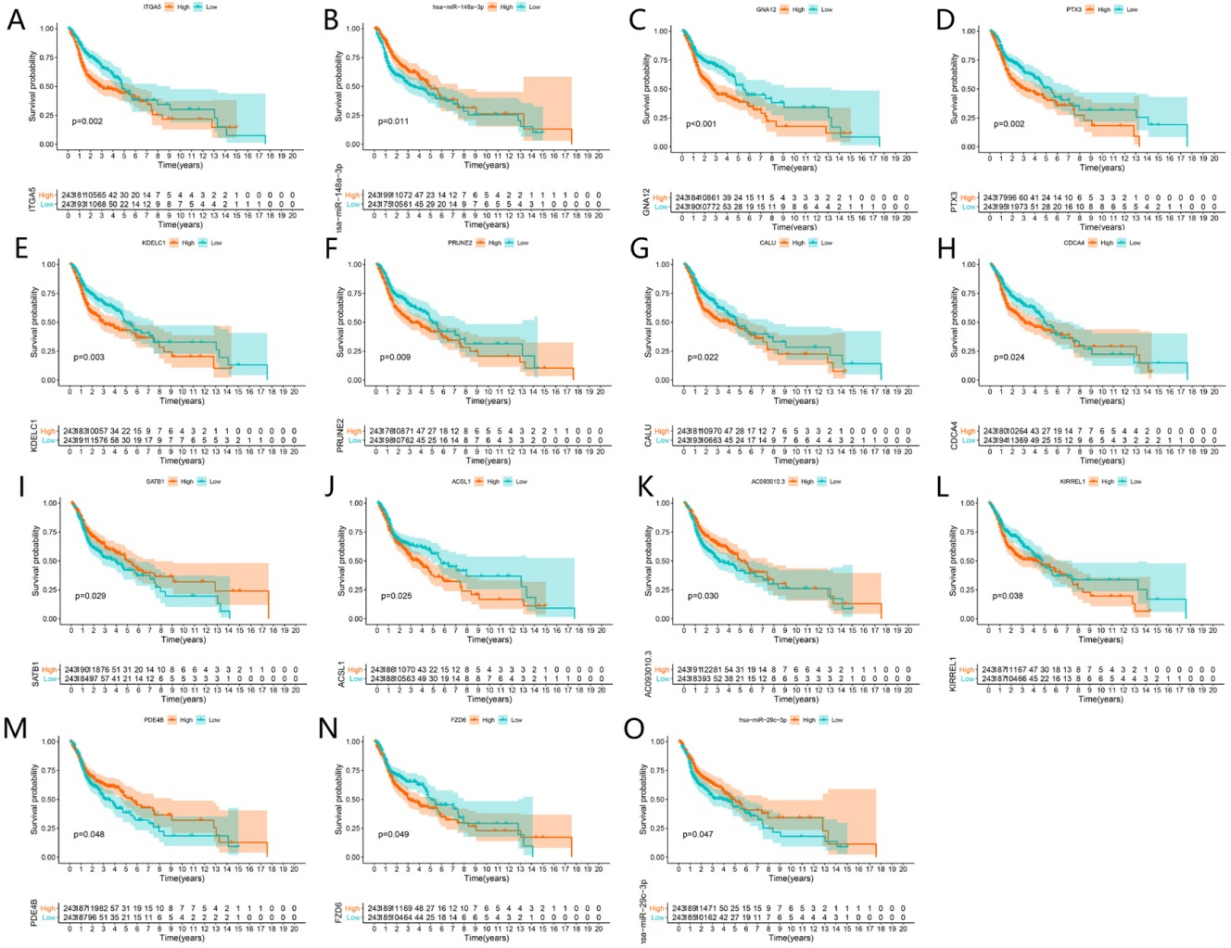
Survival analysis of significant genes in the ceRNA network. Kaplan-Meier curves of ITGA5, has-miR-148a-3p, GNA12, PTX3, KDELC1, PRUNE2, CALU, CDCA4, SATB1, ACSL1, AC093010.3, KIRREL1, PDE4B, FZD6 and has-miR-29c-3p (A-O).

A total of 4 DELs, 11 DEMs, 98 DEGs and 180 edges were included in the network (**Fig. 4A**). Subsequently, we chose lncRNA KCNQ1OT1 and its linked mRNAs and miRNAs and then built a sub-network, which contained 1 DELs, 7 DEMs, 42 DEGs and 92edges (**Fig. 4B**). Then K-M method was performed to identify prognostic RNAs in the constructed ceRNA network. The results indicated that ITGA5, has-miR-148a-3p, GNA12, PTX3, KDELC1, PRUNE2, CALU, CDCA4, SATB1, ACSL1, AC093010.3, KIRREL1, PDE4B, FZD6 and has-miR-29c-3p were significantly associated with survival (**Fig. 5A-O**).

### Construction of ceRNAs-related prognostic signature

**Figure 6:**
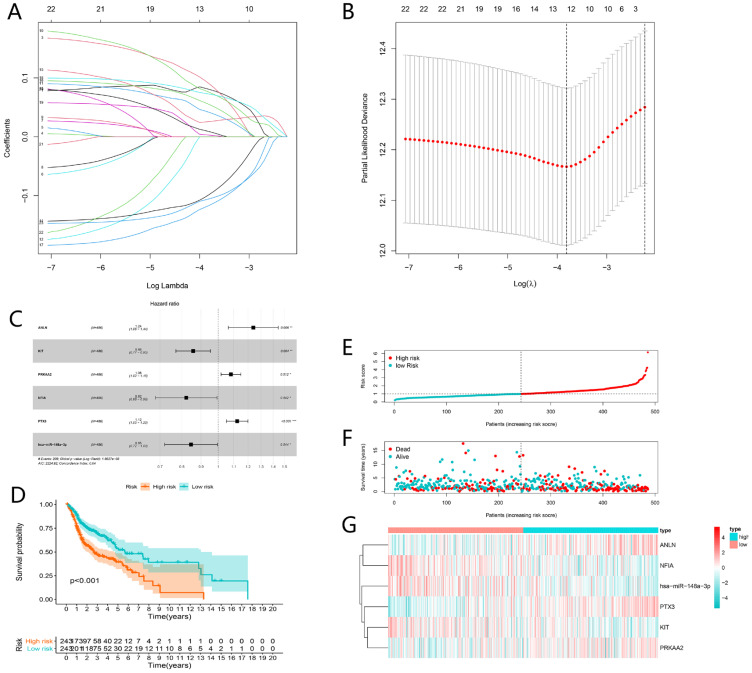
Construction and evaluation of ceRNAs-related risk signature for HNSCC. The LASSO regression analysis identified 12 genes in the ceRNA network (A). The optimal values of the penalty parameter were determined by 10-round cross-validation (B). Multivariate Cox proportional hazards regression model integrated six genes into the ceRNAs-related risk signature (C). Patients in the high-risk group indicated worse overall survival (OS) than those in the low-risk group (D). The risk curve of each patient reordered by risk score (E). The scatter plot of all patient's survival state (F). The heatmap showed the expression levels of six ceRNAs in the prognostic signature between the low-risk group and high-risk group (G).

Univariate Cox analysis was applied employing the coxph function of the “survival” package with the cut-off criteria of P-value < 0.05. In order to prevent the signature from overfitting, lasso regression was performed (**Fig. 6A**). And 12 genes were identified with the optimal adjustment parameters determined by 10-fold cross-validation (**Fig. 6B**). Ultimately, by multivariate Cox regression, 6 key biomarkers in the ceRNA network including ANLN, KIT, PRKAA2, NFIA, PTX3 and hsa-miR-148a-3p were incorporated into the ceRNAs-related signature for further study (**Fig. 6C**). The risk score was calculated by the following formula. Risk score = 0.21*ANLN-0.15*KIT+0.08*PRKAA2-0.20*NFIA+0.12*PTX3-0.17*has-miR-148a-3p (**Table 1**). The risk score was utilized to stratify patients into high- or low-risk group based on the median risk score. K-M analysis demonstrated that the high-risk group showed worse prognosis compared to the low-risk group (P < 0.001) (**Fig. 6D**), which suggested the ceRNAs-related risk signature has great prognostic prediction ability. The high-risk group also had higher risk score and mortality rate compared with the low-risk group (**Fig. 6E and F**). The heatmap showed that ANLN, PTX3, PRKAA2 were up-regulated in the high-risk group, while NF1A, miR-148a-5p and KIT were up-regulated in the low-risk group (**Fig. 6G**).

### Clinical correlation analysis and nomogram construction based on ceRNAs-related prognostic signature

**Figure 7:**
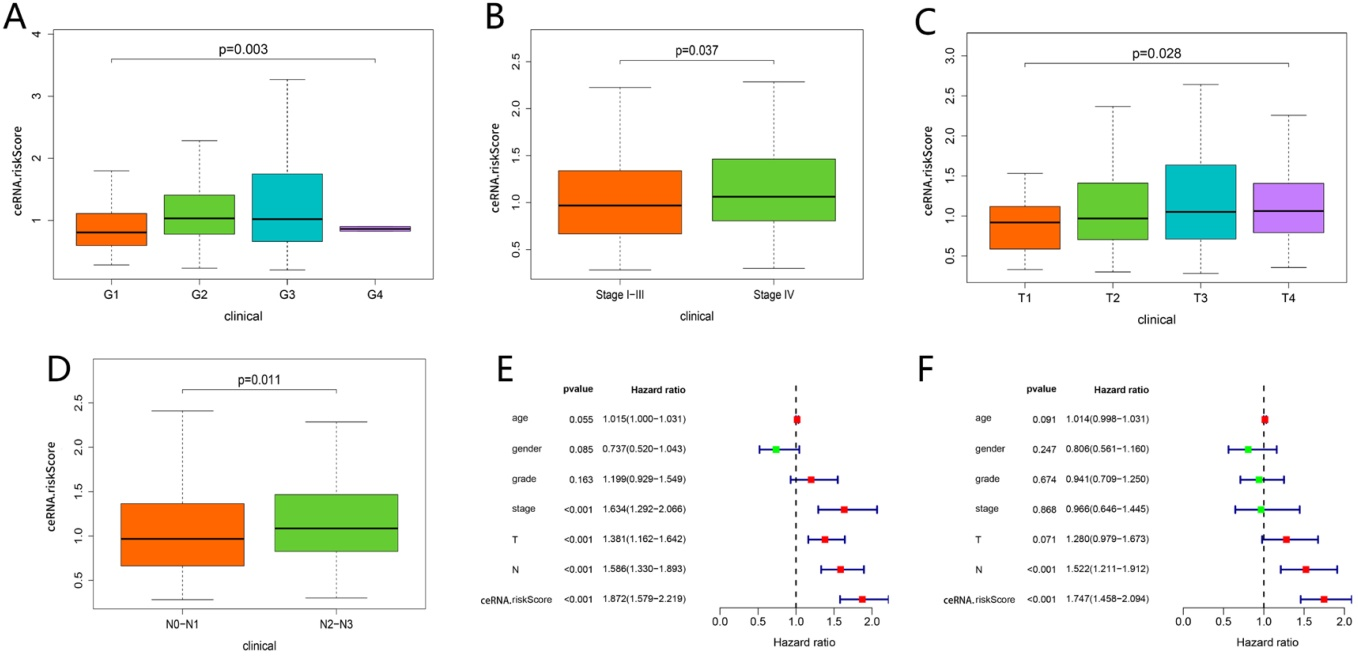
Clinical correlation analysis. Comparison of risk score among different grade (A), stage (B), tumor status (C) and lymph node metastasis degree (D). The univariate (E) and multivariate (F) Cox regression analysis of risk score, age, gender, grade, and TNM stage.

**Figure 8:**
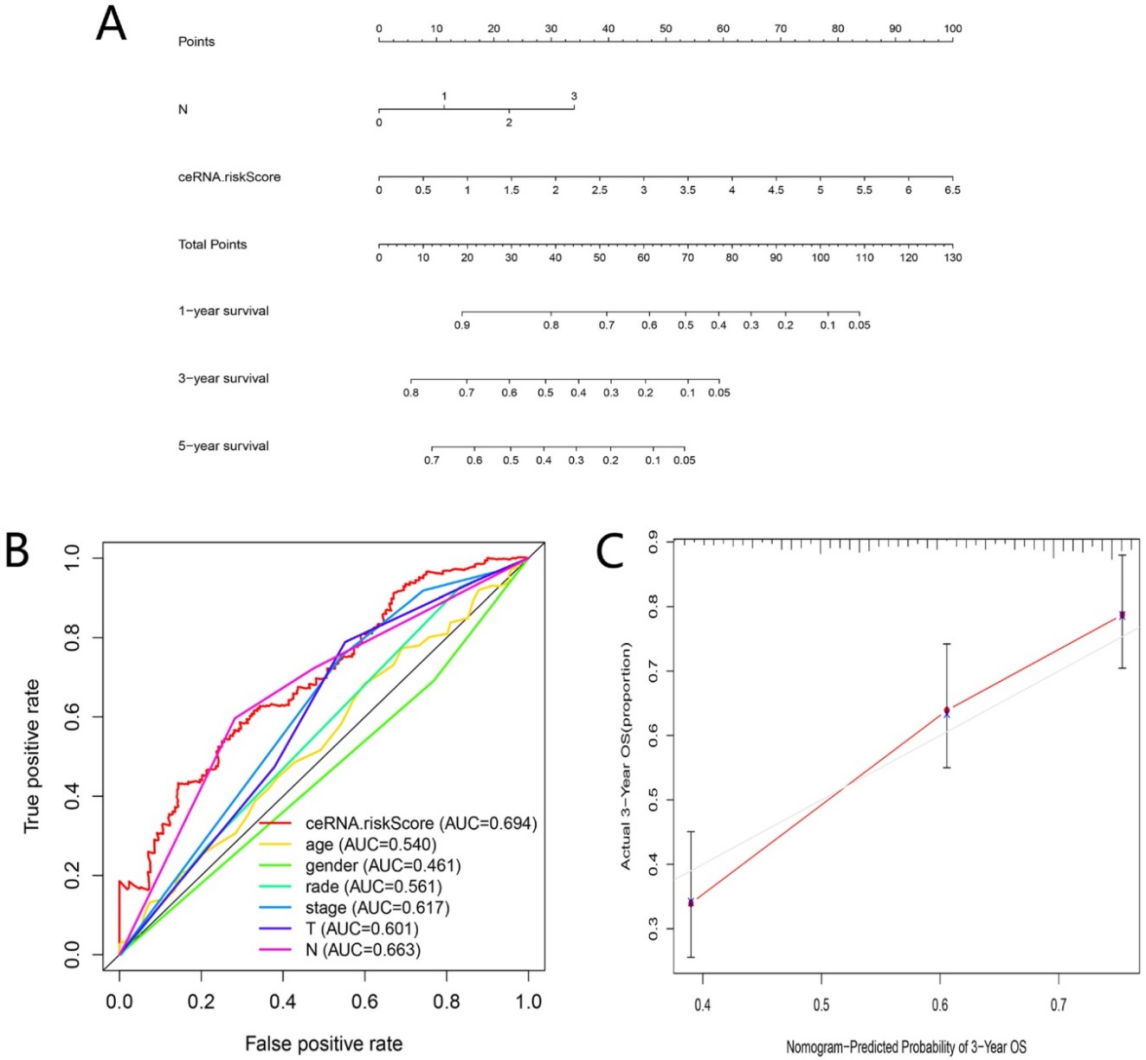
Evaluation of the ceRNAs-related signature. A nomogram based on ceRNAs-related signature and clinical characteristics (A). AUC for risk score, age, gender, grade, and TNM stage of 3-year survival according to the ROC curves (B). Calibration curve for 3-year OS (C).

As shown in** Figure 7A-D**, the risk scores among different grade exhibited statistical significance, and a higher grade was related to a higher risk score (P = 0.003). Similar results were observed in stage (P = 0.037), tumor status (P = 0.028) and lymph node metastasis degree (P = 0.011), which suggested that the ceRNAs-related signature could potentially predict malignant biological behavior of HNSCC. Univariate (HR = 1.872, 95% CI: 1.579-2.219, P < 0.001) and multivariate Cox regression analysis (HR = 1.747, 95% CI: 1.458-2.094, P < 0.001) uncovered that the ceRNAs-related signature was an independent prognostic factor in HNSCC (**Fig. 7E-F**). In order to predict the prognosis of each sample, a nomogram integrated ceRNAs-related signature and lymphatic metastasis degree was established (**Fig. 8A**). Furthermore, ROC and calibration curves exhibited an acceptable utility and discrimination for the model (AUC of 3-year was 0.694) (**Fig. 8B-C**).

### Profiles of TIICs in HNSCC

**Figure 9:**
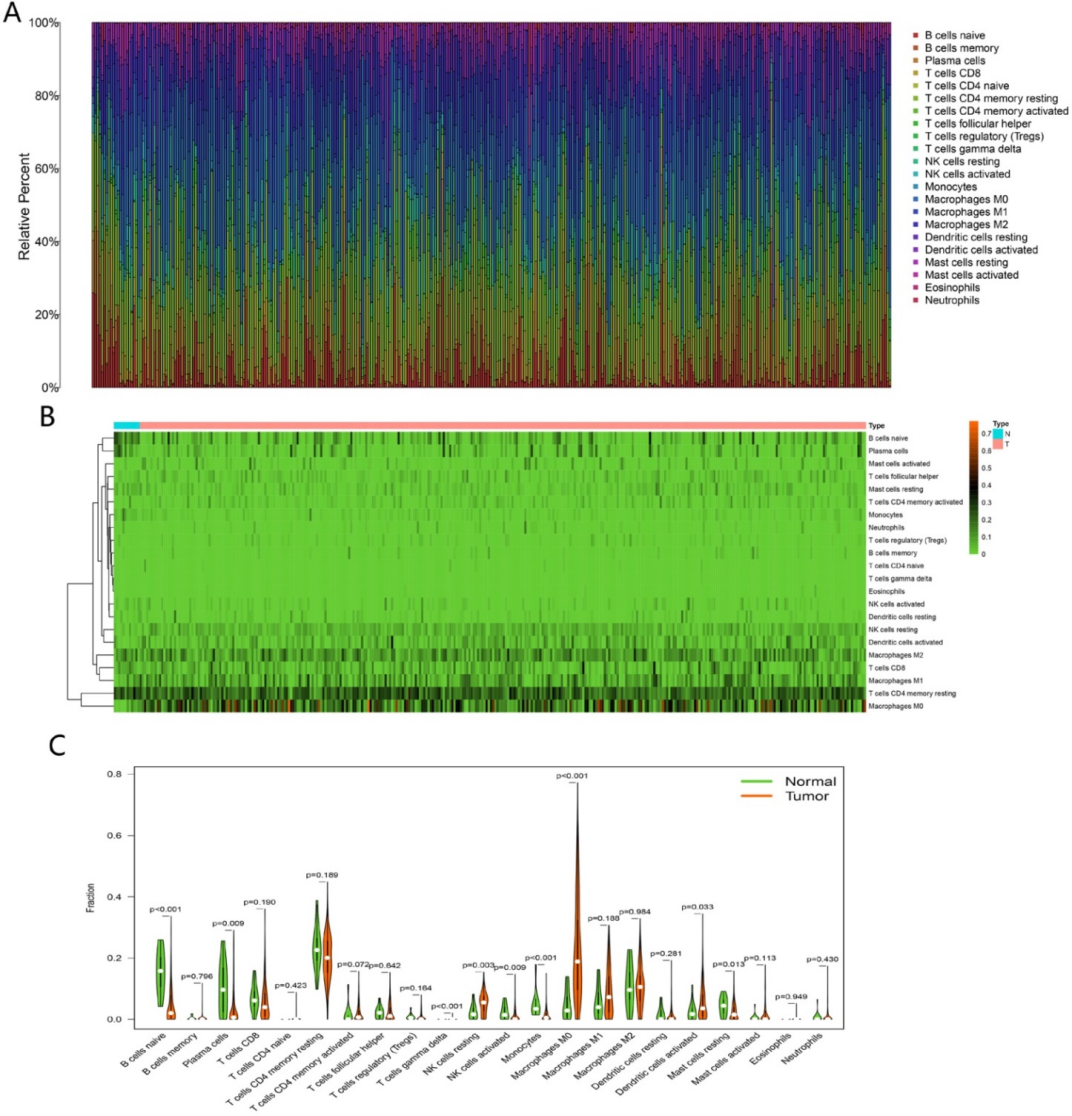
Analysis of immune infiltration. The composition of 22 immune cells estimated by CIBERSORT in HNSCC (A and B). Difference in the proportions of 22 immune cells between normal and tumor tissues (C).

The fractions of 22 TIICs were assessed by CIBERSORT algorithm, as shown in** Figure 9A-B**. To explore significantly differential distribution of TIICs in normal and tumor groups, Wilcoxon-rank sum test was conducted. The results were visualized by violin plot shown in **Figure 9C**. B cells naive, plasma cells, T cells gamma delta, NK cells resting, NK cells activated, monocytes, macrophages M0, dendritic cells activated and mast cells resting were obviously altered between two groups.

### Construction of TIICs-related prognostic signature

**Figure 10:**
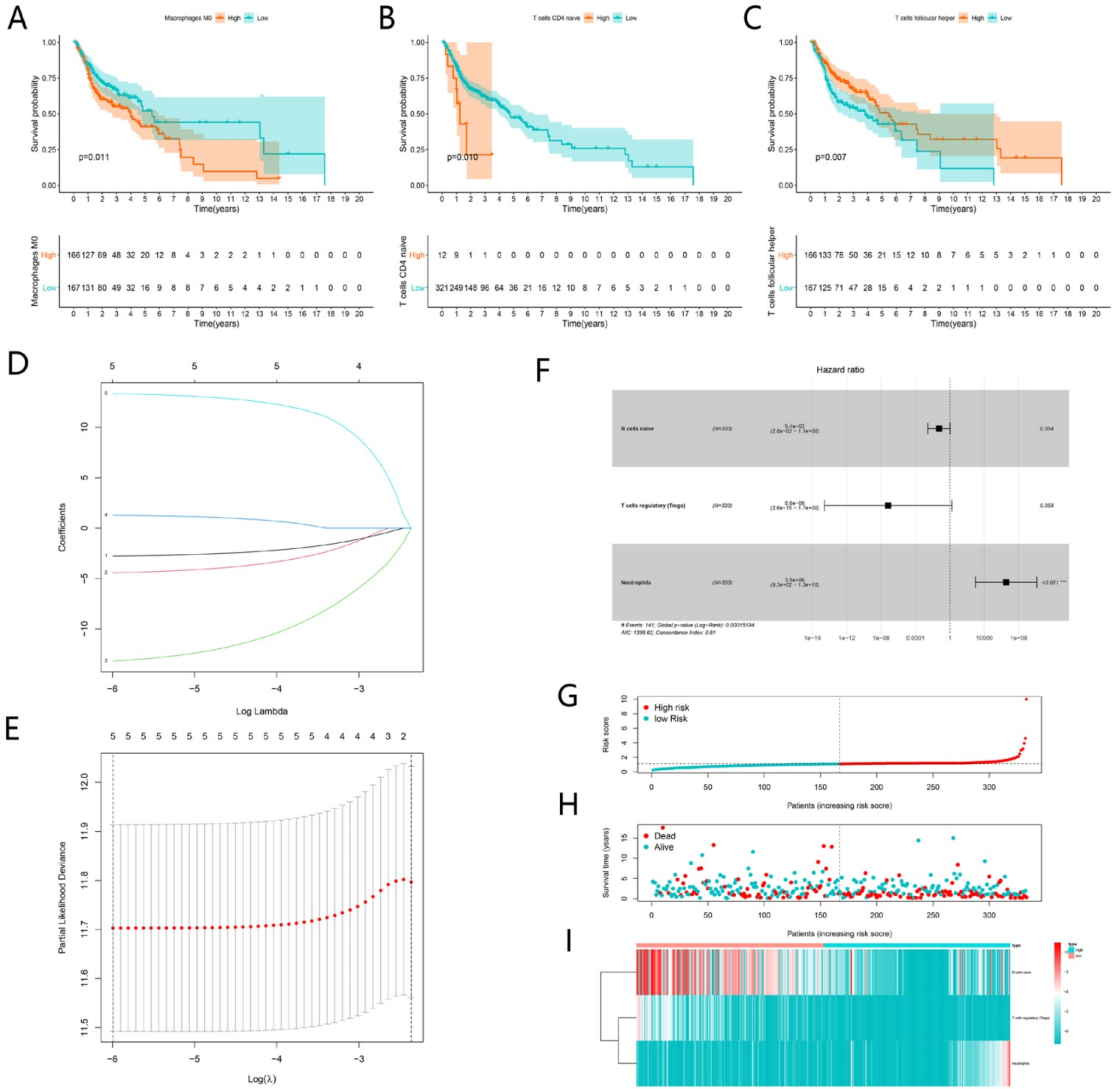
Construction of TIICs-related risk signature for HNSCC. Kaplan-Meier curves showed Macrophages M0, naive CD4 T cells and follicular helper T cells were significantly associated with survival (A-C). The LASSO regression analysis showed five TIICs were significant for modeling (D-E). Multivariate Cox proportional hazards regression model integrated three TIICs into the TIICs-related prognostic signature (F). The risk curve of each patient reordered by risk score (G). The scatter plot of all patient's survival state (H). The heatmap showed the proportion of the TIICs in the TIICs-related signature between the low-risk group and high-risk group (I).

K-M analysis was performed to identify prognostic TIICs, as shown in **Figure 10A-C**, the proportion of M0 Macrophages, naive CD4 T cells and follicular helper T cells significantly associated with survival (P < 0.05). Then 22 types of immune cell were incorporated into univariate cox regression. The results of the lasso regression indicated that the model was not overfitting (**Fig. 10D-E**). After multivariate Cox regression analysis, three TIICs including naïve B cells, T cells regulatory (Tregs) and neutrophils constituted a new TIICs-related prognostic signature (**Fig. 10F**). The risk curve and scatterplot indicated that samples in high-risk group exhibited higher risk scores and mortality rates (**Fig. 10G-H**). The fractions of three immune cells between high-risk and low-risk group were visualized by heatmap (**Fig. 10I**). In the low-risk group, the fractions of naïve B cells and Tregs were higher than those in high-risk group. However, the fraction of neutrophils behaved oppositely.

### Clinical correlation analysis and nomogram establishment based on TIICs-related signature

**Figure 11:**
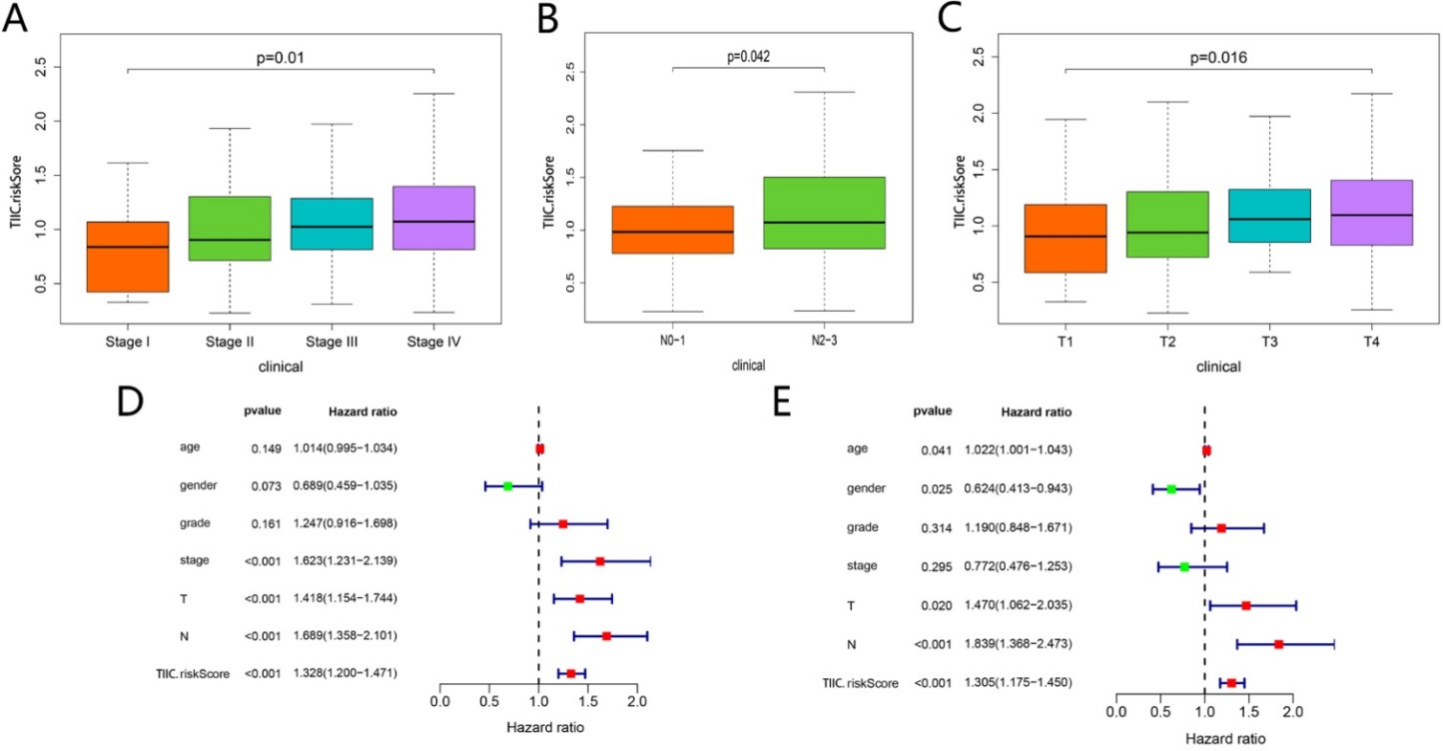
Correlational analyses between clinical information and TIIC-riskScore. Comparison of risk score among different stage (A), lymph node metastasis degree (B) and tumor status (C). The univariate (D) and multivariate (E) Cox regression analyses of risk score, age, gender, grade, and TNM stage.

**Figure 12:**
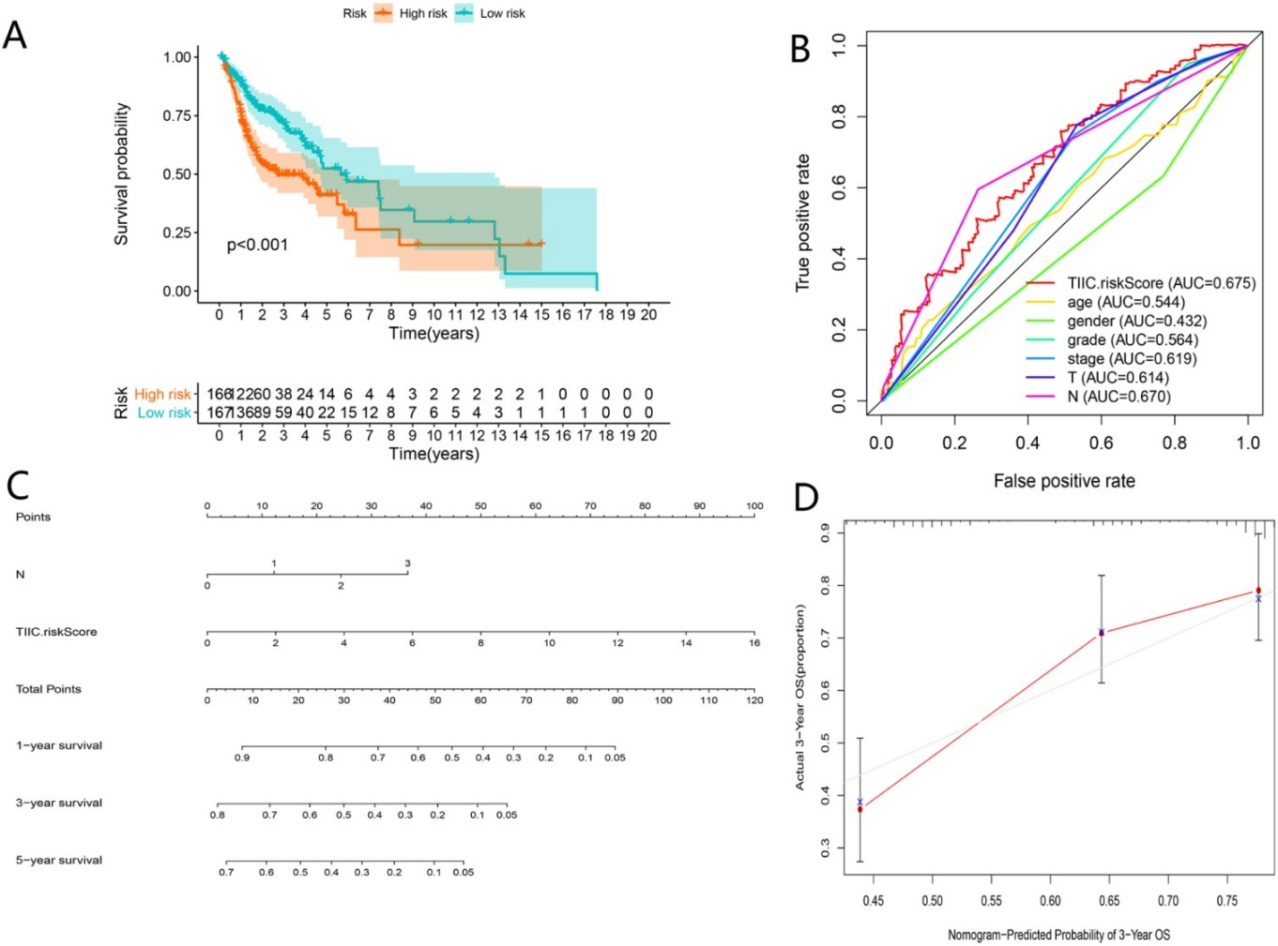
Assessment of TIICs-related prognostic signature and nomogram construction in HNSCC. Kaplan-Meier curves of the high-risk group and the low-risk group (A). ROC curves for predicting 3-year survival (B). A Nomogram integrated TIIC-riskScore and N classification (C). Calibration curve for predicting probability of 3-year OS (D).

As shown in **Figure 11A-C**, TIICs-riskScore significantly linked to stage, lymph node metastasis degree and tumor status (P < 0.05). Then univariate and multivariate Cox regression analyses showed the TIICs-related prognostic signature was an independent prognostic factor in HNSCC (P < 0.001) (**Fig. 11D-E**). K-M curve showed low-risk group had better prognosis than high-risk group (**Fig. 12A**). A nomogram integrated TIICs -related signature and lymph node metastasis degree was constructed (**Fig. 12C**). The calibration and ROC curves of 3-years exhibited acceptable accuracy and discrimination of the TIICs-related signature (**Fig. 12B, D**).

### Co-expression analysis

**Figure 13:**
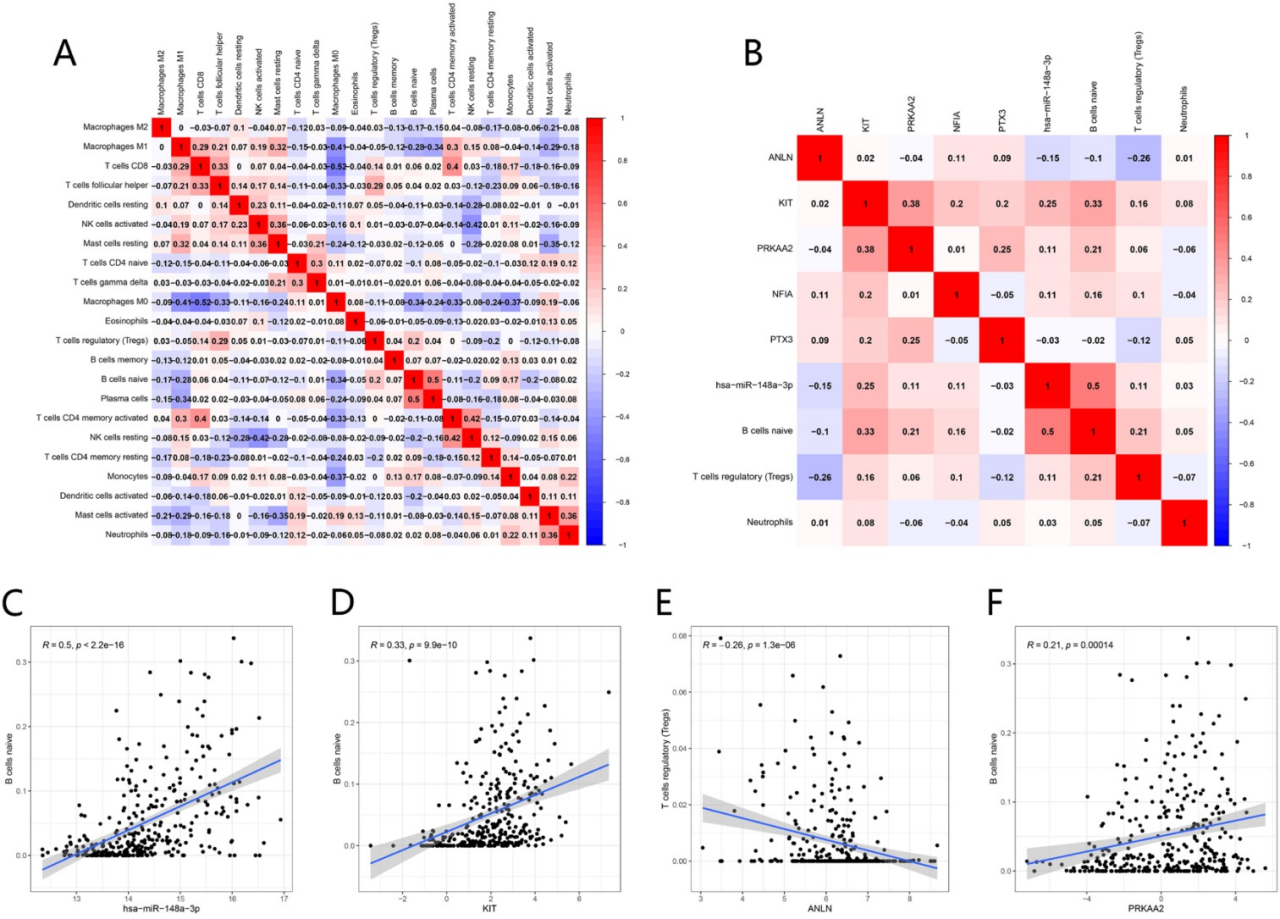
Co-expression analysis. Correlation heatmap of proportions of 22 immune cells in HNSCC (A). Correlation heatmap of prognostic TIICs and significant genes in the ceRNA network (B). has-miR-148a-3p, KTT and PRKAA2 were positively associated with naive B cells (C-D, F), while ANLN was negatively associated with T cells regulatory (Tregs) (E).

The possible correlation between each immune cell was shown in** Figure 13A**. Macrophages M0 showed a significant negative correlation to CD8 T cells (R = -0.52). Plasma cells showed a significant positive correlation to naïve B cells (R = 0.5).

The co-expression analyses between TIICs and ceRNAs were realized by Pearson correlation analysis (**Fig. 13B**). There were positive correlations between the proportion of naïve B cell and the expression level of hsa-miR-148a-3p (R = 0.5, P < 0.001), KIT (R= 0.33, P < 0.001) and PRKAA2 (R = 0.21, P < 0.001). While there was a negative association between the proportion of Tregs and the expression level of ANLN (R = -0.26, P < 0.001) (**Fig. 13C-F**).

## Discussion

HNSCC is regarded as a common malignancy with high mortality and morbidity worldwide. Although tremendous progress has made in multidisciplinary treatments, the prognosis of HNSCC currently is still unfavorable 1. Therefore, researchers are obliged to find novel biomarkers for diagnosis, prognosis and therapy. Recently, emerging evidence manifested that molecular spectrum of tumors and landscape of TIICs play a vital role in oncogenesis and progression and were frequently considered as potential prognostic biomarkers 25, 26.

That rapid development of high-throughput sequencing and bioinformatics enables researchers to identify numerous aberrant expressions of RNAs and differential fraction of immune cells between normal and tumor tissues. Different from traditional molecular and cell biological studies which emphasized a particular molecular interaction, establishment of a ceRNA network offers a more comprehensive sight of the mechanism of RNA regulation in HNSCC. In this study, we first identified prognostic ceRNAs and immune cells. Afterwards, based on these results, we established two risk signatures with great prognostic value and high accuracy and discrimination. In addition, through sub-network construction of lncRNA KCNQ1OT1 and co-expression analysis of significant ceRNAs and TIICs, we noticed a potential regulatory mechanism of KCNQ1OT1 (lncRNA), miR-148a-3P (miRNA), ITGA5 (mRNA) and naïve B cell.

LncRNA is a novel class of non-coding RNA defined as a transcript longer than 200 nucleotides, and is closely linked to tumorigenesis and cancer development in various cancers 27-29. The ceRNA hypothesis suggested a new modulation mechanism that lncRNA may inhibit miRNA function via acting as an endogenous sponge, consequently modulate the expression of target mRNA 13. In the current study, we demonstrated 116 DELs, 166 DEMs and 2219 DEGs in HNSCC samples versus their normal tissues. Through GO and KEGG analyses, we further investigated the DEGs-related function and pathways. The GO results exhibited that the functions primary contained extracellular organization, collagen fibril organization and leukocyte migration, which were related to the malignant biological behavior of HNSCC 30-32. The KEGG results showed that DEGs significantly enriched in human papillomavirus infection, PI3K-Akt signaling pathway and cytokine-cytokine receptor interaction. Human papillomavirus infection is now believed to be the primary reason for the rising incidence of HNSCC 33. PI3K/AKT signaling has been proven to play a crucial part in regulating multiple tumor cellular functions covering proliferation, growth and motility in various malignancies including HNSCC 34. These may explain that the DEGs we found in this study were significantly related to survival of HNSCC.

The ceRNA network of HNSCC was constructed based on “GDCRNATools” package for R, which contained 4 lncRNAs, 11miRNAs and 98 mRNAs. Then all RNAs in the ceRNA network were subjected to univariate and multivariate Cox analysis. Subsequently, a ceRNAs -related risk signature was established. Multivariate Cox analysis demonstrated that this signature was an independent prognostic factor of HNSCC. K-M curve and ROC curve further evaluated this signature with good predictive and prognostic value. A nomogram was also built with high accuracy evaluated by calibration curve.

In addition, according to differential expression (**Fig. S1**), survival and co-expression analysis, and RNA-RNA interaction prediction, we present a possible regulatory axis KCNQ1OT1/hsa-miR-148a-3p/ITGA5, which might closely link to the development and prognosis of HNSCC. The role of KCNQ1OT1 in various cancers was studied extensively. In oral squamous cell carcinoma (OSCC), Bao et al. found KCNQ1OT1 facilitated invasion and inhibited apoptosis via regulating miR-185-5p/Rab14 axis 35. In colorectal cancer, Duan et al. demonstrated KCNQ1OT1 was overexpressed and promoted cell growth, migration and invasion through PI3K/AKT signaling 36. In non-small cell lung cancer, Kang et al. indicated KCNQ1OT1 facilitated cell proliferation and inhibited apoptosis through modulating Mir-204-5P/ATG3 axis 37. Downregulation of miR-148a-3p closely linked the progression of several malignancies such as pancreatic cancer and gastric cancer 38, 39. Lindner et al. reported miR-148a-3p was related to drug resistance and aggressiveness of esophageal squamous cell carcinoma 40. ITGA5 was associated with tumorigenesis, migration and invasion in breast cancer 41, liver cancer 42, colorectal cancer 43 and ovarian cancer cells 44. In OSCC, ITGA5 facilitated tumor progression and regulated PI3K/AKT pathway 45. All these studies about the roles of KCNQ1OT1, miR-148a-3p and ITGA5 in various cancers were in line with our present analysis. Combined with the results of functional enrichment analysis of DEGs, we speculated that lncRNA KCNQ1OT1, as a sponge of miR-148a-3p, might regulate ITGA5 expression and modulate PI3K/Akt signaling in HNSCC. This conjecture offers a comprehensive analysis of ceRNA network and narrows the scope of research. In the future, we will focus on the relevant experimental validation *in vitro* and *in vivo*.

HNSCC is a locoregional disease that is inclined to metastasize to regional lymph nodes. Hence, comprehensive analysis of immune landscape can yield greater insight into immunity. CIBERSORT is considered to be the most accurate approach available, which not only effectively distinguishes TIICs in cancer, but also maintains consistency across different genomic data sources 46. In the current study, we utilized CIBERSORT to assess the fraction of 22 TIICs based on expression matrix of HNSCC. We discovered that the fractions of naïve B cells, Plasma cells, Monocytes, resting Mast cells and activated NK cells were significantly lower in tumor samples than normal samples (P < 0.05). Inversely, a significantly higher density of activated dendritic cells, resting NK cells, and M0 macrophages was observed in tumor (P < 0.05). We also determined that naïve B cells are positively associated with plasma cells (R = 0.5), while M0 macrophages are negatively associated with CD8 T cells (R = -0.52). Through assessing the correlation between TIICs and OS, we found patients whose M0 macrophages and naïve CD4 T cells density are higher had a shorter OS time. On the contrary, patients with higher proportion of follicular helper T cells exhibited longer OS. After lasso regression and Cox regression analysis, a TIICs-related signature composed of naïve B cells, regulatory T cells (Tregs) and neutrophils was established. This signature possessed good prognostic and predictive value based on K-M curve, ROC curve, clinical correlation analysis and Cox regression analysis. In order to offer a more individualized prediction for each patient of HNSCC, a nomogram was built on basis of TIICs-related risk signature. And the calibration curve of this nomogram showed high accuracy.

As is known to all, T-cell immune response plays a dominant role in antitumor immunity, especially in HNSCC 47. A recent study showed that HNSCC with high tumor infiltration level of FoxP3+ Tregs more often exhibited better disease-free survival 48. A meta-analysis also provided evidence that high infiltration of FoxP3+ Tregs was significantly related to worse outcomes in most malignancies, including breast, renal, cervical cancers and melanomas et al, while it correlated with favorable outcomes in esophageal, head and neck, and colorectal cancers 49. In Cox regression analysis, Neutrophil was a significant independent risk factor (P < 0.001). Neutrophils are the most abundant immune cells and are viewed as the first line of anti-infection and anti-inflammation, which also inducing tumor progression in plentiful malignancies including HNSCC 50. Neutrophils can release neutrophil elastase, matrix metalloprotease 8 (MMP8), matrix metalloprotease 9 (MMP9), vascular endothelial growth factor (VEGF), cathepsin G and proteinase-3 to degrade the extracellular matrix (ECM) and promote tumor invasion 51. Neutrophils are also capable of facilitating tumoral motility, migration and invasion. For example, HNSCC cells were verified to irritate neutrophils to release pro-inflammatory factors, which strengthened the tumor cells migration in the form of feedback 52. In tongue squamous cell carcinomas, high neutrophil infiltrating level indicated higher lymph node metastasis degree, more advanced stage and greater vulnerability to tumor recurrence 53. B cells play a pivotal role in the humoral immunity of the adaptive immune system, and respond to infected cells or tumor cells. B cells could also differentiate into memory B cells or plasma cells, which secrete immunoglobulin to bind target antigens 54. Tumor-infiltrating CD20+ B cell was demonstrated to be related to favorable outcomes in different malignancies such as breast, colorectal, gastric, non-small cell lung and head and neck cancer 55-59. The results of our study were in line with the above previous experiments.

The co-expression analysis showed that naïve B cells were positively related to miR-148a-3p (R = 0.5, P < 0.001). According to the results of Pearson correlation analysis and hypergeometric testing of ceRNA network, consequently, we speculated that interactions among miR-148a-3p, KCNQ1OT1, ITGA5 and naïve B cells were closely related to the development of HNSCC. Whereas, the specific mechanism warrants further study.

We have to admit some limitations in the current study. First, this is a retrospective study based on public database, of which clinical information was incomplete. Secondary, all data in the database derive from Western countries, so the results should be cautiously applied in Asian countries. Third, in CIBERSORT analysis, only the proportion of TIICs was considered while the location of TIICs was not considered, which may produce a certain deviation. Last but not least, this study is a bioinformatics analysis and has not been verified by cell and animal experiments. However, despite its limitations, our study firstly constructed two nomograms to predict prognosis of HNSCC based on the ceRNA network and TIICs, and applied co-expression analysis between ceRNAs and TIICs. Besides, we innovatively proposed that KCNQ1OT1, miR-148a-3p, ITGA5 and naïve B cell might closely link to the tumorigenesis and progression of HNSCC. Further biological researches should be performed to validate our results. Notably, we are considering if exosomes secreted by tumor cells contain ceRNAs, which interact with TIICs and promote tumorigenesis and progression in HNSCC.

## Conclusion

In this study, we established two prognostic signatures and their nomograms with excellent prognostic value and utility based on the ceRNA network and TIICs. These two prognostic signatures may provide comprehensive clinical information for clinicians to make individualized treatment decisions. Particularly, with co-expression analysis between ceRNAs and TIICs, we speculated that the interactions among KCNQ1OT1, hsa-miR-148a-3p, ITGA5 and naïve B cells might closely correlate with the initiation and progression of HNSCC.

## Supplementary Material

Supplementary figure S1.

## Figures and Tables

**Table 1:** Six prognostic ceRNAs identified from multivariate Cox regression analysis

ceRNAs	coef	HR	HR.95L	HR.95H	*p*-value
ANLN	0.215	1.240	1.064	1.444	0.006
KIT	-0.154	0.858	0.772	0.953	0.004
PRKAA2	0.078	1.081	1.018	1.149	0.012
NFIA	-0.196	0.822	0.681	0.993	0.042
PTX3	0.116	1.123	1.051	1.200	0.001
hsa-miR-148a-3p	-0.167	0.847	0.720	0.995	0.044
