# Supplementary figures and images for: Identification novel prognostic signatures for Head and Neck Squamous Cell Carcinoma based on ceRNA network construction and immune infiltration analysis
Source: Int J Med Sci. 2021 Jan 19;18(5):1297–311. doi: 10.7150/ijms.53531 (PMC7847625; doi:10.7150/ijms.53531)

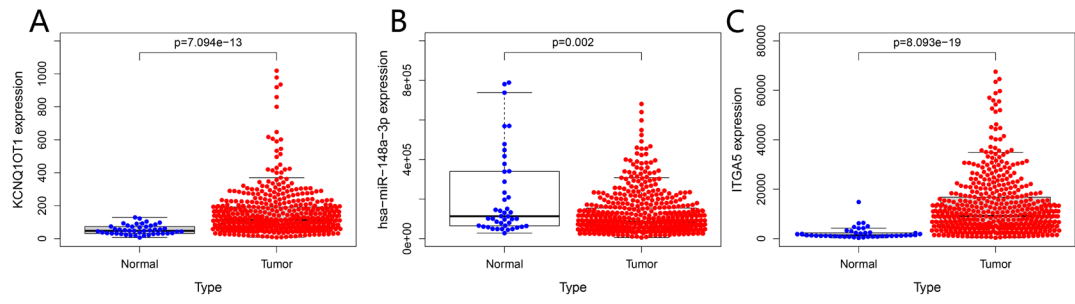

Supplement: Supplementary file 1 — Supplementary figure S1. [file ijmsv18p1297s1.pdf]
